# Society Meetings

**Published:** 1901-01

**Authors:** 


### SOCIETY MEETINGS.

THE Niagara Falls Academy of Medicine met at the office of Dr. W. R. Campbell, on Monday evening, December 3, 1900, at 8.45 p.m. Dr. C. G. Leo-Wolf read a paper.

THE Physicians' Club at its last meeting was entertained at the Genesee Hotel by Dr. D. P. Doyle. Cases of interest were reported by Drs. E. M. Dooley, J. Henry Dowd and J. J. Finerty. The essayist was Dr. James S. Porter, whose subject was The Smallpox Epidemic of 1888-1889.

The Third Pan-American Congress that was to have been held at Havana, December 26-29, 1900, has been postponed to February 4-8, 1901. While yellow fever was still somewhat prevalent, though subsiding, it was not thought prudent to hold the meeting. The success of the Congress is already assured. More than 100 papers are now on the program and others are constantly being added. Dr. Ramon Guiteras, 75 West 55th Street, New York, is the Secretary.

---

The Buffalo Academy of Medicine held meetings during the month of November and December, 1900, as follows:

*Section on Obstetrics.*—Tuesday evening, November 27th. Program: Cocainisation of the spinal cord, by means of lumbar puncture in laboi, Wm. Ridgeley Stone, of the New York City Hospital; Discussion by Marcel Hartwig and Matthew D. Mann. Appendicitis complicating pregnancy, with report of a case, Eugene A. Smith. The meeting of this section announced in the program for Tuesday, December 25th (Christmas Day), is postponed to Thursday evening, January 3, 1901.

*Section on Surgery.*—Tuesday evening, December 4th. Program: The closure and dressing of abdominal wounds, Stephen Y. Howell. Treatment of septic peritonitis, Eugene A. Smith.

*Section on Medicine.*—Tuesday evening, December 11. Program: Cyclic or periodic vomiting in childhood, Charles G. Stockton. The treatment of disease locally by hot air, Prescott LeBreton.

*Section on Ophthalmology, Otology, Rhinology and Laryngology.*—Monday evening, December 17th. Program: The etiology and treatment of concomitant strabismus; a reconsideration, Alvin A. Hubbell. The influence of the vertical muscles in the production of asthenopia, Arthur G. Bennett.

*Section on Pathology.*—Tuesday evening, December 18th. Program: Some notes on globulin and albumose, as found in urine, A. L. Benedict. Acute infection, Eugene Wasdin, Marine surgeon.

---

The Medical Society of the County of Erie will hold its eightieth annual meeting at the Y.M.C.A. building, corner of Mohawk and Pearl Streets, at 10 o'clock a.m., Tuesday, January 8, 1901. At this meeting eight delegates to the Medical Society of the State of New York will be elected and other important business transacted.

A program is in preparation of which the following are some of the features: A Country Health Officer, Dr. E. H. Ballou, President, Gardenville; Legislation in reference to tuberculosis, Dr. J. H. Pryor; A brief résumé of the grosser animal nature and its application in medicine, Dr. Geo. N. Jack, Depew, N.Y.

---

THE Erie County Medical Association held a meeting for organisation Thursday, December 20, 1900, at the Buffalo Library rooms. The call, which contained grandiose statements with reference to the New York State Medical Association, likewise several so-called material inducements to become members of the new county organisation, was signed by the following-named physicians: DeLancey Rochester, chairman; Arthur G. Bennett, secretary; Chas. G. Stockton, Alvin A. Hubbell, Chas. A. Wall, W. H. Thornton, committee.

---

THE Medical Society of the State of New York will hold its 95th annual meeting in the City Hall, at Albany, January 29, 30 and 31, 1901, under the presidency of Dr. A. M. Phelps, of New York City.

The temporary program lately issued contains an excellent group of papers, by men of distinction, both from within and without the state. Among the guests from other states who will participate in the proceedings are Dr. Charles A. L. Reed, of Cincinnati, president of the American Medical Association; Dr. L. S. McMurtry, of Louisville; Dr. George Ben Johnston, of Richmond; Dr. Wm. E. B. Davis, of Birmingham, Dr. James F. W. Ross, of Toronto; Dr. J. Collins Warren, of Boston; Dr. Thomas Roddick, of Montreal; Dr. John B. Murphy, of Chicago; Dr. Merrill B. Ricketts, of Cincinnati, and Dr. Victor C. Vaughan, of Ann Arbor. The names from Buffalo on the program are Dr. Henry R. Hopkins, Dr. Herman Mynter, Dr. John H. Pryor, Dr. Wm. C. Krauss, Dr. Lucien Howe and Dr. William Warren Potter.

On Wednesday morning, January 30th, at 8.15 o'clock, a demonstration clinic, conducted by Drs. Albert Vander Veer, Samuel B. Ward and Willis G. Macdonald, of the Albany Hospital staff, will be held at the Albany hospital.

Wednesday evening a reception will be held by the president from 6.30 to 7.30, and the annual dinner will be served at the Hotel Ten Eyck at 8 o'clock.

There will be two discussions during the meeting. The first, on Wednesday morning, will begin with a paper, entitled, A Contribution to pelvic infection in women, by Charles A. L. Reed, Cincinnati; discussed by J. F. W. Ross, Toronto, Ont.; L. S. McMurtry, Louisville, Ky.; W. E. B. Davis, Birmingham, Ala.; Bache McE. Emmet, New York; William M. Polk, New York; George M. Edebohls, New York; James Clifton Edgar, New York; James P. Boyd, Albany. This will be followed by a report of five cases of ovarian tumors with twisted pedicles, by George Ben Johnston, Richmond.

The president's address, at 12.15, on Hernia will close the morning session.

The second discussion will open the Wednesday afternoon session, at 2 o'clock, and will begin with a paper on Surgery of the spleen, by J. Collins Warren, Boston, Mass; discussed by Albert Vander Veer, Albany; Charles McBurney, New York; Robert F. Weir, New York; Robert T. Morris, New York; Thomas Roddick, Montreal, Can.

The following papers will then be read: The pathology and diagnosis of intestinal obstruction, J. B. Murphy, Chicago; Inguinal hernia, Merrill B. Ricketts, Cincinnati; Toxin of the colon bacillus, Victor C. Vaughn, Ann Arbor, Mich.

Communications relating to the presentation of papers or to any changes in the preliminary program, should be sent to the business committee: Frank Van Fleet, chairman, 63 East 79th St., New York City; J. B. Ransom, Dannemora; or F. H. Parker, Auburn.

